# Mental health in Canadian children and adolescents during COVID-19 pandemic: the role of personality and, coping and stress responses

**DOI:** 10.3389/fpsyg.2023.1190375

**Published:** 2023-06-19

**Authors:** Anahita Shokrkon, Elena Nicoladis

**Affiliations:** ^1^Department of Psychology, University of Alberta, Edmonton, AB, Canada; ^2^Department of Psychology, University of British Columbia, Kelowna, BC, Canada

**Keywords:** COVID-19, pandemic, mental health, personality, big 5 personality traits, extroversion, neuroticism

## Abstract

In December 2019, the Coronavirus Disease (COVID-19) pandemic first emerged in China and quickly spread to other countries. Previous studies have shown that the COVID-19 pandemic and the consequences have negatively impacted the mental health of adults. Individual differences such as personality could contribute to mental health. Furthermore, coping and responses to stress may affect an individual’s response to the pandemic. In the past, studies have only investigated this relationship in adults. In the current study, we examine how personality traits (using the Five-Factor Model as our framework) and Coping and Response to COVID-19 stress are related to the mental health of Canadian children and adolescents during the pandemic. Using parent reports of 100 preschoolers and 607 6–18-year-old children, we performed multiple regression analysis to explore how personality traits predict the effects of COVID-19 on mental health. The results showed that personality traits are associated with the mental health of Canadian youth during the COVID-19 pandemic. In preschoolers, Neuroticism and Agreeableness predicted the most mental health problems, and in 6-18-year-old children, Extraversion negatively predicted the most mental health problems. Also, Openness to Experience was the weakest predictor of mental health status in Canadian youth. These findings could be useful in understanding children’s responses to the COVID-19 pandemic and could assist public health services delivering mental health services specifically tailored to children’s personalities during and after this pandemic.

## Introduction

The Novel Coronavirus (COVID-19) epidemic started in China in December 2019 and was declared a global pandemic in early 2020, by World Health Organization (2020). Several studies have documented that the public’s mental health has deteriorated since the outbreak of COVID-19 in early 2020 (Hossain et al., 2020; Jiao et al., 2020; Gadermann et al., 2021; Kumar and Nayar, 2021). According to Statistics Canada, based on a Survey on COVID-19 and Mental Health, one in four (25%) Canadians expressed symptoms of depression, anxiety, or post-traumatic stress disorder (PTSD) in spring 2021, compared with one in five (21%) in fall 2020 (Statistic Canada, 2021).

Only a relatively small number of children have been physically affected by the disease (She et al., 2020), with most showing only mild symptoms compared to adults (Nigg et al., 2020). Nevertheless, some of the public health policies, like the containment measures, could negatively affect children’s mental health. The COVID-19 pandemic has brought significant educational and social disruptions to children around the world. For instance, in Canada, school closures affected 5.7 million children and youth attending elementary and secondary school (Statistic Canada, 2021). Even when schools remained open, students’ experiences of schooling were changed dramatically. For instance, in order to prevent the spread of COVID-19, curriculum delivery methods have changed, social distancing measures have been implemented during classes and recess and mask-wearing has been mandated across Canada (Vaillancourt et al., 2021). Moreover, many parents were forced to work from home due to the pandemic and although some children could benefit from increased interactions with them, many have experienced increased levels of emotional distress (Sprang and Silman, 2013; Xie et al., 2020). Having to stay at home could also disturb children’s sleep/wake cycles, and physical exercise activities, and lead to excessive use of technology (Xie et al., 2020). The pandemic has also been associated with an increase in family economic stressors and parental unemployment, which could result in short- and long-term mental health effects in children (Costello et al., 2003). Furthermore, domestic violence and emotional, physical, or sexual abuse are more likely to occur in a situation like the pandemic (Ramaswamy and Seshadri, 2020; Ali et al., 2021).

Children’s development has also been impacted by the drastic changes in all aspects, particularly the interruptions to the development of skills that are fundamental for optimum growth and wellness (Araújo et al., 2021). For instance, children and youth are spending less time interacting with peers and adults compared with before the pandemic (McNamara, 2021), resulting in immediate adverse consequences (Moore et al., 2020), such as negative effects on cognitive development (Deoni et al., 2022), socio-emotional development (Egan et al., 2021), academic performance (Davies and Aurini, 2021) and mental health (Kang et al., 2021).

Not all individuals have been equally affected by the pandemic. Several studies have shown that individual differences such as personality traits could predict differences in psychosocial and mental health outcomes, and the well-being of adults before the pandemic (Albuquerque et al., 2012; Strickhouser et al., 2017; Bucher et al., 2019) and also during the pandemic (Proto and Zhang, 2021; Shokrkon and Nicoladis, 2021). Moreover, personality traits could influence the coping style individuals select in different stressful situations (van Berkel, 2009) as coping is a dynamic process that changes with time as a consequence of varying demands and perceptions of the situation (Moos and Holahan, 2003). Nonetheless, a limited number of studies have explored the contribution of personality and coping to the impacts of the pandemic on the well-being of children and adolescents. Personality is defined as a set of mental structures and adaptive strategies acquired throughout life via socialization and the further expression of temperament within individuals (Rothbart et al., 2000). In this study, we are testing how personality traits, using the Five-Factor Model (FFM; McCrae and Costa, 1987), are associated with the mental health of children and adolescents during the COVID-19 pandemic in Canada. The FFM is one of the most widely recognized summaries of human personality traits (Eysenck, 1992; Goldberg, 1993) consisting of the five dimensions of Neuroticism (or Emotional Instability vs. Stability), Extroversion (vs. Introversion), Openness to Experience (or unconventionality), Agreeableness (vs. Antagonism), and Conscientiousness (or Constraint vs. Disinhibition) (Goldberg, 1992). The FFM framework has been shown to be valid and applicable to children as young as preschool age (Abe and Izard, 1999; Halverson et al., 2003; Asendorpf and Denissen, 2006; Grist and McCord, 2010). Understanding how personality affects children’s mental health during the COVID-19 pandemic may assist public health services to implement services tailored to each child’s personality.

Also, alongside the personality traits, we are interested in examining the contribution of Coping and Response to Stress on the mental health of Canadian youth as coping could have a central role in determining the impact of the pandemic (Buheji et al., 2020), as how one responds to stress could have significant long-term and immediate consequences (Corbett et al., 2021). Coping consists of cognitive and behavioral strategies used to manage stress (Biggs et al., 2017). There are two types of coping strategies: adaptive and maladaptive (Compas et al., 2017). Adaptive strategies including primary control engagement coping (in which one attempts to modify the stressor directly or modify one’s response to the stressor, for example, by solving problems) and secondary control engagement coping (which focuses on adjusting to the problem, for example, using cognitive reorganization) have been found to significantly reduce the risk of negative mental health outcomes (Carver et al., 1989; Connor-Smith et al., 2000; Rosenberg et al., 2011). In contrast, maladaptive strategies including disengagement coping (which involves attempts to suppress arousal, for example, using avoidance), involuntary engagement coping (which involves involuntary emotional and physiological stress, for example, using distracting thoughts and sympathetic arousal), and involuntary disengagement coping (which involves attempts to disengage from one’s emotions, for example using through emotional numbing) have been linked to adverse mental health outcomes (Compas et al., 1997; Connor-Smith et al., 2000; Matthews et al., 2016). Therefore, the coping behaviors and strategies used by children and adolescents play an important role in maintaining an appropriate mental health adjustment in a situation like the current pandemic (Idoiaga et al., 2020), therefore, we will investigate their contribution to children’s mental health.

### The present study

In this study, we are interested in to see how personality traits contribute to mental health of children from preschool to adolescence. Online questionnaires were sent to parents of 2–18-year-old children living across Canada using Qualtrics, a survey platform, distributed via social networks and from the email listings of the University of Alberta. Prior to participating in the study, parents consented through Qualtrics to a question asking “Do you wish to continue the survey, if you do your consent to participate is implied,” with two options of “I consent” and “I do not wish to continue.” The study was approved by the Research Ethics Board at the University of Alberta (Pro00100751). The data used in this study is available in the Figshare repository for other researchers to use. Participation in our study was voluntary and ten random participants received a $50 gift card of their choice.

In our study, we analyzed the data of children under 6 and over 6 separately for two reasons: (1) the personality measurement we used differed for the two groups and (2) we believe children being in school vs. out of school could have various impacts. For instance, containment measures and policies were different for daycares and schools and also, school-aged children are often able to use technology independently to contact friends remotely.

## Materials and methods

### Participants

A sample of 100 parents of preschool children (80 mothers, 18 fathers, and 2 other caregivers; mean age = 33.75 years [SD = 5.73; range 18 to 48 years]) were recruited for this study who completed parent reports of questionnaires during June and July 2021. The children of participants were 52 males, and 48 females; mean age = 3.52 years [SD = 1.14; range 2 to 6 years]. To make sure our preschooler’s study is not under powered, we used GPower software application. This power analysis is undertaken to determine the minimum sample size required. The required power was set at 1- β = 0.80. Level of significance was set at α = 0.05. Effect size was kept at the range value of 0.15 and the number of predictors is taken as 12. Power analysis revealed that in order to achieve, power of 0.80, a total sample size of *n* = 44 is needed for our study.

Also, 607 parents of 6-18-year-old children participated in our study (350 mothers, 257 fathers; mean age = 37.56 years [SD = 3.47; range 21 to 58 years]) and completed parent reports of questionnaires. The children of participants were 307 males, and 300 females; mean age = 12.55 years [SD = 3.47; range 6 to 18 years]. All participants were required to: (1) reside in Canada; and (2) consent to participate.

### Measures

#### Mental health

Mental health was assessed using parents’ reports on the Strengths and Difficulties Questionnaire (SDQ; Goodman, 1997). We used the age-appropriate versions of SDQ for parents of preschoolers and 6–18-year-old children. The SDQ asks questions about positive and negative characteristics displayed by the child in the past 6 months in five categories: Emotional Symptoms (e.g., often unhappy, downhearted), Conduct Problems (e.g., fights with other children), Hyperactivity/Inattention (e.g., constantly fidgeting or squirming), Peer Relationship Problems (e.g., tends to play alone) and Prosocial Behavior (e.g., considerate of other people’s feelings). There are five items in each subscale, and the parent/care giver rates each item as either: Never = 0, Somewhat True = 1, or Certainly True = 2. The Total Difficulties score is generated by adding up the values of the first four scales, leading to a possible score between 0 and 40, where higher scores indicate an increasing level of behavioral difficulties. A total SDQ score of 17 or higher is considered abnormal. In a study by Croft et al. (2015), all sub-scales showed acceptable internal reliability of subscales ranging from ω = 0.66 (Peer Relationship Problems) to ω = 0.83 (Hyperactivity/Inattention) in preschool children. Another study on 6-17-year-old Canadian children found evidence for the factorial validity and reliability of the parent-rated SDQ and acceptable internal consistency ranging from 0.79 to 0.88 for the subscales (Hoffmann et al., 2020).

### Personality

#### Preschoolers’ measurement

The M5–PS–35 is a five-factor personality questionnaire that has been validated for use in preschool populations (Grist et al., 2012). The M5-PS-35 includes items such as “is friendly towards peers” (Extraversion), “loves to help people” (Agreeableness), “completes tasks successfully” (Conscientiousness), “is afraid of many things” (Neuroticism), and “adapts to new activities” (Openness to Experience). Each question uses a 5-point Likert-type scale, ranging from Inaccurate to Accurate. Grist et al. (2012) showed that the revised and shortened version shows strong construct validity and improved internal reliability, internal consistency values are E = 0.77; A = 0.90; C = 0.87; N = 0.79; O = 0.71.

#### 6–18-year-olds measurement

Personality of 6–18-year-old children was measured by parents’ reports on the Big Five Questionnaire for Children (BFQ-C) which is a 65-item questionnaire that measures the Big Five in children and adolescents (Barbaranelli et al., 2003). The traits are Extraversion (e.g., “I easily make friends”), Agreeableness (e.g., “I trust in others”), Conscientiousness (e.g., “I like to keep all my school things in order”), Neuroticism/Emotional instability (e.g., “I easily get angry”) and Openness/Intellect (e.g., “I easily learn what I study at school”). Items are scored on a five-point Likert scale ranging from 1 = almost never to 5 = almost always. For each factor, individual item scores are combined to yield a total score. A study by Vreeke and Muris (2012) found that parent report on the BFQ-C was found to have good alphas for Extraversion (0.79), Agreeableness (0.87), Conscientiousness (0.88), Neuroticism (0.86), and Openness (0.86).

### Coping and response to stress

Coping and Responses to Stress Questionnaire (RSQ) COVID-19 (Connor-Smith et al., 2000), a multidimensional questionnaire, is adapted to specific stressors or domains of stress, in this case, the COVID-19 pandemic. There are 57 items categorized into five factors (Connor-Smith et al., 2000). For each item, participants are asked: *How much do you do this?* On a scale of 1 to 4: 1 (Not at All), 2 (A Little), 3 (Some), and 4 (A lot). The five total factors include three types of *coping*: Primary Control Engagement Coping: (i.e., emotional expression, emotion regulation, and problem-solving); Secondary Control Engagement Coping (i.e., acceptance, cognitive restructuring, distraction, and positive thinking); and Disengagement Coping (i.e., avoidance, denial, and wishful thinking). The RSQ includes two types of involuntary responses to *stress*: Involuntary Engagement (i.e., emotional arousal, impulse action, intrusive thoughts, physiological arousal, and rumination) and Involuntary Disengagement (i.e., cognitive interference, emotional numbing, escape, and inaction). Each of the five factors—Primary Coping, Secondary Coping, Disengagement Coping, Involuntary Engagement, and Involuntary Disengagement—is calculated as a ratio score of the total stress response items endorsed. Therefore, Primary Coping, for example, represents the propensity of an individual to use this coping style relative to the four other factors. The RSQ has demonstrated excellent internal consistency, test–retest reliability, and convergent and construct validity (Compas et al., 2017; Coiro et al., 2021).

### Demographic variables

All participants were asked to provide the following demographic information: parents’ age, their current job status and if there has been a change in their income over the last 2 months if they had pre-existing mental health issues, children’s age and gender, and the number of children in the family and birth order of children.

Our participants were also asked specific questions about their experiences with the COVID-19 pandemic, for example, if they or anyone living in their household were diagnosed with COVID-19, whether they experienced domestic conflicts as a consequence of the pandemic, how the pandemic interfered with their social interactions, if the loss of childcare services affected them and if the children experienced any issues with their siblings. We reasoned that children’s mental health could be affected by the personal experiences people have with the COVID-19 pandemic. Table 1 (preschoolers) and Table 2 (6–18-year-old children) provide additional demographic information.

**Table 1:** Preschool sample demographics characteristics.

Demographics	Options	Percentage
Child’s gender		
	Male	52%
	Female	48%
Job Status		
	Not employed	14%
	Temporary/Part-time Employment	13%
	Full-time Employment	30%
	Student	43%
Social interactions		
	Not Affected	10%
	Somewhat Affected	50%
	Largely Affected	40%
Sibling Issues		
	Did not have sibling issues	89%
	Did have sibling issues	11%

**Table 2:** 6–18-year-olds sample demographics characteristics.

Demographics	Options	Percentage
*Income change*		
	Yes, it has decreased	51.7%
	Yes, it has increased	39.5%
	No change	8.7%
*Job status*		
	Not employed	31.6%
	Temporary/Part-time Employment	28.7%
	Full-time Employment	33.9%
	Student	5.8%
Domestic conflict		
	Yes	85.5%
	No	14.5%
		
Social interactions		
	Not Affected	69.5%
	Affected	30.5%
Sibling issues		
	Did not have sibling issues	53.2%
	Did have sibling issues	46.8%

### Statistical analyses

Following are the results of all analyses conducted in SPSS (Version 28). In order to investigate the relationship between personality traits with mental health (positive and negative attributes), first, we calculated Pearson correlation coefficients between personality traits on one hand and Total Difficulty scores, Emotional Symptoms, Hyperactivity/Inattention, Peer Relationship Problems, and Prosocial Behaviors on the other, as well as demographic factors. The variables with at least one significant association with outcome variables are presented in Table 3 (preschoolers) and Table 4 (6–18-year-old children).

**Table 3:** Means, standard deviations, and correlations between demographics variables, coping and stress responses, and personality traits in preschoolers.

	MEAN (SD)	1	2	3	4	5	6	7	8	9	10	11	12	13	14	15	16	17	18	19	20
1. Child Gender 1.1 Male Female	–	- REF																			
2. Job Status 2.1 Part-time No Job	–	-02 REF	–																		
3. Social Interactions 3.1 Largely No Change	–	-11 REF	0.02	–																	
4. Sibling Issue 4.1 No Yes	–	0.08 REF	0.05	−0.21**	–																
5. Primary Control Coping	0.31 (0.14)	0.20	0.06	0.07	0.23*	–															
6. Secondary Control Coping	0.32 (0.13)	−0.01	0.07	−0.06	0.11	0.02	–														
7. Disengagement Coping	0.13 (0.12)	−0.10	−0.08	−0.09	−0.21*	−0.46**	−0.43**	–													
8. Involuntary Engagement Coping	0.10 (0.10)	−0.05	−0.14	0.01	−0.13	−0.42**	−0.47**	0.04	–												
9. Involuntary Disengagement Coping	0.09 (0.07)	−0.03	−0.04	−0.01	−0.00	−0.54**	−0.39**	0.14	0.49**	–											
10. Openness	4.17 (0.63)	0.10	0.03	−0.06	0.13	0.17	0.39**	−0.05	−0.32**	−0.29**	–										
11. Extraversion	4.29 (0.75)	0.14	0.05	0.03	0.00	0.17	0.42**	−0.15	−0.39**	−0.42**	0.52	–									
12. Neuroticism	2.84 (0.83)	−0.11	−0.03	0.12	−0.16	−0.21*	−0.23*	−0.01	0.41**	0.23*	−0.34**	−0.36**	–								
13. Agreeableness	3.28 (0.66)	0.01	0.20*	−0.16	0.30**	0.27**	0.15	−0.14	−0.26*	−0.18	0.24*	−0.00	−0.38**	–							
14. Conscientiousness	3.66 (0.63)	0.16	0.12	−0.04	0.19*	0.26*	0.18	−0.12	−0.26*	−0.31**	0.41**	0.26**	−0.40**	0.58**	–						
15. Emotional Symptoms	2.58 (2.55)	−0.18	−0.01	−0.01	−0.03	−0.28**	−0.24*	0.01	0.48*	0.32*.	−0.31**	−0.42**	0.65**	−0.20*	−0.37**	–					
16. Conduct Problems	2.31 (1.44)	−0.01	−0.10	0.20*	−0.36**	−0.21*	−0.23*	0.05	0.30**	0.24*	−0.22*	−0.12	0.49**	−0.62**	−0.42**	0.39**	–				
17. hyperactivity/inattention	5.65 (2.43)	−0.04	−0.25*	0.10	0.17	−0.14	−0.08	0.05	0.25*	0.06	−0.14	0.13	0.23*	−0.58**	−0.58**	0.20*	0.42**	–			
18. Peer relationship problems	4.12 (1.64)	−0.19*	−0.01	−0.04	−0.05	−0.10	−0.17	0.01	0.27*	0.21*	−0.25*	−0.32**	−0.30**	−0.19	−0.30**	0.50**	0.27**	0.13	–		
19. Prosocial behavior	7.74 (2.10)	0.11	0.07	0.08	0.33**	0.23*	0.24*	−0.27**	−0.17	−0.24*	0.33**	0.37**	−0.17	0.24*	0.39**	−0.15	−0.22*	−0.21*	−0.22*	–	
20. Total difficulties score	14.66 (5.68)	−0.16	−0.14	0.07	−0.21*	−0.27*	−0.25*	0.04	0.47**	0.29**	−0.33**	−0.25**	0.60**	−0.55**	−0.61**	0.78**	0.69**	0.66**	0.64**	−0.28**	

**Table 4:** Means, standard deviations, and correlations between demographics variables, coping and stress responses and personality traits in 6–18-year-old children.

	MEAN (SD)	1	2	3	4.1	4.2	5	6	7.1	8	9	10	11	12	13	14	15	16	17	18	19	20	21	22	21
1. Parent’s age	37.56 (5.98)																								
2. Child’s age	12.55 (3.47)	0.11**																							
3. Income Change Decrease No change	–	−0.06 REF	0.03																						
4. Job Status 4.1. Part-time 4.2. Student No Job	–	−0.04 0.01	0.01 –0.14**	0.14** –0.15**	– −0.15**																				
5. Domestic issues Yes No		−0.35** REF	0.07	0.25**	0.14**	−0.38**																			
6. Social interactions Yes No change	–	−0.07 REF	−0.00	0.09*	0.04	−0.13**	0.20**																		
7. Sibling issue Yes No		−0.10* REF	0.07	−0.02	−0.00	−0.07	0.14**	0.02																	
8. Primary control coping	0.16 (0.03)	0.17**	−0.03	−0.09*	−0.04	0.30**	−0.46**	−0.12**	−0.14**																
9. Secondary control coping	0.21 (0.03)	0.23**	−0.03	−0.13**	−0.08*	0.24**	−0.52**	−0.08*	−0.14**	0.30**															
10. Disengagement coping	0.15 (0.02)	−0.09*	0.01	0.01	0.01	−0.01	0.12**	0.03	0.02	−0.31**	−0.26**														
11. Involuntary engagement coping	0.25 (0.03)	−0.13**	0.02	0.13**	0.06	−0.26**	0.46**	0.10*	0.16**	−0.51**	−0.66**	−0.07													
12. Involuntary disengagement coping	0.20 (0.03)	−0.23**	0.04	0.08*	0.05	−0.29**	0.46**	0.08*	0.11**	−0.57**	−0.56**	−0.01	0.24**												
13. Openness	40.47 (6.34)	0.15**	−0.11**	−0.11**	−0.09*	0.28**	−0.47**	−0.12**	−0.12**	0.39**	0.35**	−0.12**	−0.32**	−0.36**											
14. Extraversion	40.98 (6.62)	0.17**	−0.16**	−0.13**	0.06	0.30**	−0.49**	−0.10**	−0.13**	0.37**	0.33**	−0.13**	−0.26**	−0.36**	0.45**										
15. Neuroticism	38.36 (5.92)	−0.15**	0.00	0.03	0.00	−0.27**	0.28**	0.03	0.12**	−0.23**	−0.32**	0.04	0.32**	0.22**	−0.13**	−0.21**									
16. Agreeableness	41.22 (6.96)	0.18**	−0.09*	−0.19**	−0.09*	0.43**	−0.55**	−0.16**	−0.16**	0.39**	0.37**	−0.06	−0.34**	−0.40**	0.47**	0.42**	−0.22**	-							
17. Conscientiousness	39.91 (6.10)	0.09*	−0.00	−0.07	−0.08*	0.15**	−0.26**	−0.09*	−0.16**	0.27**	0.19**	−0.06	−0.20**	−0.22**	0.29**	0.25**	−0.15**	0.33**							
18. Emotional symptoms	4.64 (2.06)	−0.14**	0.02	0.06	−0.01	−0.21**	0.31**	0.10*	0.11**	−0.19**	−0.35**	0.06	0.30**	0.22**	−0.15**	−0.21**	0.20**	−0.19**	−0.15**	-					
19. Conduct problems	4.34 (2.22)	−0.23**	0.11**	0.17**	0.13**	−0.37**	0.56**	0.06	0.12**	−0.38**	−0.35**	0.11**	0.28**	0.40**	−0.31**	−0.42**	0.22**	−0.47**	−20**	0.25**					
20. Hyperactivity/inattention	4.99 (2.02)	−0.09*	−0.00	0.00	0.00	−0.13**	0.11**	0.02	0.01	−0.16**	−0.17**	0.06	17**	0.12**	−0.17**	−0.05	0.18**	−0.11**	−0.23**	0.16**	0.12**				
21. Peer relationship problems	4.70 (2.24)	−0.20**	0.01	0.18**	0.12**	−0.28**	0.49**	0.11**	0.03	−0.30**	−0.33**	0.05	0.30**	0.30**	−0.32**	−0.35**	0.20**	−0.36**	−0.14**	0.23**	0.33**	0.09*			
22. Prosocial behavior	5.51 (2.11)	0.16*	−0.09*	−0.19**	−0.16**	0.31**	−0.41**	−0.10*	−0.05	0.28**	0.26**	−0.01	−0.26**	−0.28**	0.33**	0.31**	−0.14**	0.44**	0.16*	−0.10*	−0.32**	−0.08*	−0.24**	-	
23. Total difficulties score	18.66 (5.44)	−0.26**	0.05	0.16**	0.10**	−0.40**	0.59**	0.12**	0.11**	−0.41**	−0.47**	0.11**	0.42**	0.42**	−0.38**	−0.42**	0.32**	−0.46**	−0.28**	0.64**	0.69**	0.52**	0.67**	−0.30**	-

A hierarchical multivariate regression model was then used to assess the relationship between independent variables and outcome variables. Among demographic variables and Coping and Response to COVID-19 Stress factors, those significantly associated with the dependent variables (Total Difficulty scores, Conduct Problems, Emotional Symptoms, Hyperactivity/Inattention, Peer Relationship Problems, and Prosocial Behaviors) during bivariate analyses were entered into the first and the second models of the hierarchical regression models. The five Coping and Response to COVID-19 Stress factors, Primary Coping, Secondary Coping, Disengagement Coping, Involuntary Engagement, and Involuntary Disengagement were entered in the second block in order to control for potential confounding variables (Table 5 for preschoolers and Table 6 for 6–18-year-old children show the final block of the three hierarchical regression analyses).

**Table 5:** Hierarchical regression analysis (standardized beta weights) of personality traits in relation to SDQ subscales, controlled for demographics and coping and stress responses variables for preschoolers.

	Total Difficulty			Emotional			Conduct			Hyperactivity			Peer			Prosocial		
	Beta	95% CI		Beta	95% CI		Beta			Beta	95% CI		Beta	95% CI		Beta	95% CI	
Gender Male Female	N/A			N/A			N/A			N/A			−0.10 REF	−0.98	0.31	N/A		
Job status part-time No Job	N/A			N/A			N/A			−0.10 REF	−1.92	0.40	N/A			N/A		
Social interactions largely No Change	N/A			N/A			0.11 REF	−0.16	0.93	N/A			N/A			N/A		
Sibling issue No Yes	0.09 REF	−0.73	2.86	N/A			−0.20* REF	−1.76	−0.14	N/A			N/A			0.31* REF	0.80	3.55
Primary control coping	0.01	−7.16	8.39	−0.05	−4.70	2.83	0.05	−1.64	2.80	N/A			N/A			0.01	−7.04	8.00
Secondary control coping	−0.00	−8.76	8.62	0.01	−3.93	4.44	−0.04	−2.88	1.93	N/A			N/A			0.13	−1.80	5.67
Disengagement Coping	N/A			N/A			N/A			N/A			N/A			N/A		
Involuntary engagement Coping	0.20*	0.27	23.76	0.18	−0.87	10.50	0.01	−3.02	3.45	0.18*	0.14	8.67	0.08	−2.75	5.35	0.08	−2.69	5.52
Involuntary disengagement Coping	−0.00	−15.80	15.58	0.02	−6.86	8.35	0.14	−0.1.68	7.19	N/A			0.00	−5.16	5.24	0.17	−1.80	9.24
Openness	0.02	−1.53	2.05	0.06	−0.62	1.18	−0.00	−0.51	0.47	0.02	−0.64	0.84	−0.06	−8.17	0.49	0.10	−0.48	1.20
Extraversion	0.02	−1.36	1.78	−0.10	−1.11	0.41	0.04	−0.34	0.52	0.30**	0.35	1.67	−0.18	−1.01	0.19	0.34**	0.28	1.76
Neuroticism	0.32**	1.02	3.58	0.54**	1.07	2.30	0.21*	0.03	0.74	−0.03	−0.64	0.45	0.12	−0.23	0.74	0.08	−0.38	0.81
Agreeableness	−0.21*	−3.61	−0.16	0.15	−0.23	1.43	−0.47**	−1.53	−0.56	−0.27**	−1.78	−0.25	−0.01	−0.71	0.62	0.09	−0.51	1.13
Conscientiousness	−0.30**	−4.63	−0.88	−0.18	−1.62	0.15	0.03	−0.42	0.59	−0.47**	−2.6	−1.04	−0.12	−1.03	0.38	0.13	−0.40	1.33

N/A: not applicable.

**p < 0.01;

*p < 0.05.

Total difficulty: *R*^2^ = 0.06 for block 1 (*F*(1,100) = 5.40; *p* < 0.05); Δ*R*^2^: 0.20 for block 2 (*F*change(5,100) = 5.73; *p* < 0.001). Δ*R*^2^ = 0.34 for block 3 (*F*change(10,100) = 11.34; *p* < 0.001). Emotional Problems: *R*^2^ = 0.23 for block 1 (*F*(4,100) = 6.04; *p* < 0.001); Δ*R*^2^: 0.28 for block 2 (*F*change(9,100) = 8.92; *p* < 0.001). Conduct: *R*^2^ = 0.20 for block 1 (*F*(2,100) = 10.78; *p* < 0.001); Δ*R*^2^: 0.08 for block 2 (*F*change(6,100) = 5.28; *p* < 0.001). Δ*R*^2^ = 0.26 for block 3 (*F*change(11,100) = 7.79; *p* < 0.001). Hyperactivity/inattention: *R*^2^ = 0.05 for block 1 (*F*(1,100) = 4.89; *p* < 0.05); Δ*R*^2^: 0.05 for block 2 (*F*change(2,100) = 4.87; *p* < 0.05). Δ*R*^2^ = 0.44 for block 3 (*F*change(7,100) = 13.81; *p* < 0.001). Peer problems: *R*^2^ = 0.00 for block 1 (*F*(1,100) = 0.53; *p* > 0.05); Δ*R*^2^: 0.08 for block 2 (*F*change(5,100) = 1.37; *p* > 0.05). Δ*R*^2^ = 0.14 for block 3 (*F*change(10,100) = 2.12; *p* < 0.05). Prosocial: *R*^2^ = 0.16 for block 1 (*F*(1,100) = 0.16; *p* < 0.001); Δ*R*^2^: 0.07 for block 2 (*F*change(5,100) = 4.92; *p* < 0.001). Δ*R*^2^ = 0.16 for block 3 (*F*change(10,100) = 4.72; *p* < 0.001).

**Table 6:** Hierarchical regression analysis (standardized beta weights) of personality traits in relation to SDQ subscales, controlled for demographics and coping and stress responses variables for 6–18 year-old children.

	Total Difficulty			Emotional			Conduct			Hyperactivity			Peer			Prosocial		
	Beta	95% CI		Beta	95% CI		Beta			Beta	95% CI		Beta	95% CI		Beta	95% CI	
1. Parent’s age	−0.05	−0.10	0.01	−0.03	−0.03	0.02	−0.03	−0.04	0.01				−0.02	−0.03	0.01	0.03	−0.01	0.03
2. Child’s age	N/A			N/A			0.04	−0.01	0.06	N/A			N/A			−0.03	−0.06	0.02
3. Income change Decrease Increase	0.01 REF	−0.88	0.61	N/A			0.02	−0.19	0.40	N/A			0.05	−0.10	0.56	−0.07	−0.62	0.00
4. Job status 4.1. Part-time 4.2. Student No Job	−0.01 –0.13** REF	−5.01	−1.61	N/A − 0.09*	−1.66	−0.06	0.02 –0.10**	−0.19 –1.78	0.45 –0.28	N/A − 0.08	−1.56	0.04	0.03 –0.05	−0.19 –1.30	0.52 0.31	−0.07 0.08*	−0.66 0.02	0.01 1.58
5. Domestic issues Yes No	0.24** REF	2.44	5.36	0.11*	0.03	1.38	0.28**	1.20	2.46	−09	−1.21	0.15	0.26**	1.05	2.44	−0.10*	−1.32	−0.00
6. Social interactions Yes No change	−0.01 REF	−0.84	0.61	0.04	−0.14	0.54	N/A			N/A			0.01	−0.29	0.40	0.00	−0.31	0.34
7. Sibling issue Yes No	−0.01 REF	−0.80	0.55	0.04	−0.14	0.49	0.03	−0.16	0.42	N/A			N/A			N/A		
Primary control coping	−0.63*	−225.07	−1.88	0.02	−7.04	9.68	−0.30	−70.83	26.00	−0.06	−12.89	4.07	−0.01	−9.20	7.73	−0.01	−8.79	7.37
Secondary control coping	−0.82*	−233.44	−11.90	−0.19**	−18.86	−2.77	−0.30	−66.30	29.79	−0.10	−13.97	2.34	−0.01	−9.06	7.73	−0.07	−12.18	3.32
Disengagement coping	−0.42	−214.68	8.10	N/A			−0.15	−63.29	33.32	N/A			N/A			N/A		
Involuntary engagement coping	−0.60	−207.81	14.81	0.08	−3.15	13.27	−0.30	−67.91	28.63	0.01	−7.72	8.92	0.06	−4.28	12.33	−0.09	−13.99	1.77
Involuntary disengagement coping	−0.54	−207.94	13.72	0.00	−8.31	9.30	−0.14	−58.91	37.23	−0.03	−11.55	6.55	0.03	−6.00	11.84	−0.08	−14.10	2.83
Openness	−0.03	−0.09	0.03	0.04	−0.01	0.04	0.05	−0.00	0.04	−0.12**	−0.07	−0.01	−0.05	−0.05	0.01	0.06	−0.06	0.05
Extraversion	−0.08*	−0.13	−0.00	−0.05	−0.04	0.01	−0.13**	−0.07	−0.01	0.10*	0.00	0.06	−0.10*	−0.06	−0.00	0.08	−0.00	0.05
Neuroticism	0.07*	0.00	0.13	0.04	−0.01	0.04	0.01	−0.02	0.03	0.11**	0.01	0.06	0.03	−0.01	0.04	0.02	−0.01	0.03
Agreeableness	−0.05	−0.10	0.01	0.05	−0.01	0.04	−0.15**	−0.08	−0.02	0.04	−0.01	0.04	−0.06	−0.05	0.00	0.23**	0.04	0.10
Conscientiousness	−0.09**	−0.13	−0.02	−0.06	−0.05	0.00	0.00*	−0.02	0.02	−0.19**	−0.09	−0.03	0.03	−0.01	0.04	−0.02	−0.03	0.02

N/A: not applicable

**p < 0.01;

*p < 0.05.

Total difficulty: *R*^2^ = 0.39 for block 1 (*F*(7,607) = 52.89; *p* < 0.001); Δ*R*^2^: 0.05 for block 2 (*F*change(12,607) = 37.87; *p* < 0.001). Δ*R*^2^ = 0.02 for block 3 (*F*change(17,607) = 29.39; *p* < 0.001). Emotional Problems: *R*^2^ = 0.11 for block 1 (*F*(5,607) = 15.36; *p* < 0.001); Δ*R*^2^: 0.05 for block 2 (*F*change(9,607) = 12.58; *p* < 0.001). Δ*R*^2^ = 0.01 for block 3 (*F*change(14,607) = 8.60; *p* < 0.001). Conduct: *R*^2^ = 0.34 for block 1 (*F*(7,607) = 43.63; *p* < 0.001); Δ*R*^2^: 0.03 for block 2 (*F*change(12,607) = 28.21; *p* < 0.001). Δ*R*^2^ = 0.03 for block 3 (*F*change(17,607) = 22.30; *p* < 0.001). Hyperactivity/inattention: *R*^2^ = 0.02 for block 1 (*F*(3,607) = 5.44; *p* = 0.001); Δ*R*^2^: 0.03 for block 2 (*F*change(7,607) = 4.66; *p* < 0.001). Δ*R*^2^ = 0.06 for block 3 (*F*change(12,607) = 6.22; *p* < 0.001). Peer problems: *R*^2^ = 0.25 for block 1 (*F*(6,6,007) = 33.08; *p* < 0.001); Δ*R*^2^: 0.01 for block 2 (*F*change(10,607) = 21.06; *p* < 0.001). Δ*R*^2^ = 0.02 for block 3 (*F*change(15,607) = 15.16; *p* < 0.001). Prosocial: *R*^2^ = 0.20 for block 1 (*F*(7,607) = 21.64; *p* < 0.001); Δ*R*^2^: 0.02 for block 2 (*F*change(11,607) = 15.01; *p* < 0.001). Δ*R*^2^ = 0.05 for block 3 (*F*change(16,607) = 13.32; *p* < 0.001.

We entered the (correlated) demographic variables and Coping and Response to COVID-19 Stress variables into the first and second blocks to control for them, as we reasoned that those demographic variables and Coping and Response to COVID-19 Stress play a critical role in determining the impact of the pandemic on children. Finally, after controlling for demographics and Coping and Response to COVID-19 Stress variables, the five personality traits were entered into the model.

## Results

### Preschool children

The means, standard deviations, and correlations between personality traits, Coping and Response to COVID-19 Stress variables, and SDQ subscales are presented in Table 3. Extraversion, Agreeableness, Openness to Experience, and Conscientiousness are all positively correlated with each other and negatively correlated with Neuroticism, except for Extraversion and Agreeableness.

As can be seen in Table 5, Openness to Experience is not related to any of the SDQ subscales when controlling for demographic factors and Coping and Response to COVID-19 Stress variables. Extraversion is positively and significantly related to Hyperactivity/Inattention and Prosocial Behavior. Neuroticism is positively and significantly related to Total Difficulties score, Emotional Symptoms, and Conduct Problems. Agreeableness was negatively and significantly related to Total Difficulties score, Conduct problems, and Hyperactivity/Inattention. Conscientiousness is negatively and significantly related to Total Difficulties score, and Hyperactivity/Inattention.

On top of the demographics and Coping and Response to COVID-19 Stress variables, the personality traits, explained 60% of the variance of total difficulty score, 51% of Emotional Symptoms, 54% of Conduct Problems, 54% of Hyperactivity/Inattention, 22% of Peer Relationship Problems, and 39% Prosocial Behavior.

### 6–18-Year-old children

The means, standard deviations, and correlations between personality traits, Coping and Response to COVID-19 Stress variables, and SDQ subscales are presented in Table 4. Extraversion, Agreeableness, Openness to Experience, and Conscientiousness are all positively correlated with each other and negatively correlated with Neuroticism.

As can be seen in Table 6, Openness to Experience is negatively related to Hyperactivity/Inattention when controlling for demographic factors and Coping and Response to COVID-19 Stress variables. Extraversion was negatively and significantly related to Total Difficulty score, Conduct Problems, and Peer Relationship Problems and also positively related to Hyperactivity/Inattention. Neuroticism was positively and significantly related to total difficulty score and Hyperactivity/Inattention. Agreeableness was negatively and significantly related to Conduct Problems and positively related to Prosocial behaviors. Conscientiousness was negatively and significantly related to Total Difficulty score, and Hyperactivity/Inattention.

On top of the demographics and Coping and Response to COVID-19 Stress variables, the personality traits, explained 46% of the variance of the Total Difficulty score, 17% of Emotional Symptoms, 40% of Conduct Problems, 11% of Hyperactivity/Inattention, 28% of Peer Relationship problems, and 27% Prosocial behavior.

## Discussion

### Preschool children

The results of preschoolers’ data show that on top of the demographic variables and Coping and Response to COVID-19 Stress, personality traits predicted substantial variance in the effects of COVID-19 on the mental health of preschoolers in Canada which will be discussed in greater detail in the following section.

Among the five Coping and Response to COVID-19 Stress factors, Involuntary Engagement Coping (or stress reactivity) was the only variable that was significantly associated with SDQ subscales of the Total Difficulties score and Hyperactivity/Inattention (see Table 5). Involuntary Engagement Coping is characterized by involuntary emotional and physiological stress, for example through intrusive thoughts, rumination, impulsive actions, and physiological arousal, out of one’s control. Previous studies have also shown associations between maladaptive stress response of Involuntary Engagement Coping with greater psychopathology (Singer et al., 2000) and mental issues such as anxiety, depression, internalizing problems, and aggression (Wolff et al., 2009; Dufton et al., 2010; Blöte et al., 2022). Moreover, children who are highly reactive experience greater illness rates in situations of increased stress (Boyce et al., 1995) similar to the COVID-19 situation happening now.

### Mental health and personality traits in preschoolers

#### Neuroticism

Among the big 5 personality traits, Neuroticism predicted the most SDQ difficulty subscales (Total Difficulty, Emotional Symptoms, and Conduct Problems) among these preschoolers. Neuroticism is a personality trait characterized by a disposition to experience negative emotions which manifests itself through feelings of anxiety, anger, sadness, and tension (John et al., 2008). In Study 1, Neuroticism predicted the Total Difficulties score, namely the sum of Emotional Symptoms, Conduct Problems, Hyperactivity/Inattention, and Peer Relationship Problems scores. Neuroticism has long been linked to psychopathology and evidence suggests that Neuroticism reflects a common vulnerability contributing to the development and maintenance of a variety of mental illnesses (Sauer-Zavala et al., 2017). Generally, Neuroticism has been found to be a risk factor for developing emotional disorders such as depression and anxiety (Lahey, 2009; Agh-Yousefi and Maleki, 2011; Andrés et al., 2016). To explain the vulnerability to emotional problems, some studies have shown that anxiety sensitivity, intolerance of uncertainty and worry, and rumination could be vulnerability markers related to Neuroticism (Sexton et al., 2003; Broeren et al., 2011). Also, Neuroticism could also be a predictor of conduct disorder as according to Eysenck’s biological theory of personality, Neuroticism is associated with higher psychobiological reactivity in the face of frustration and greater sympathetic arousal (Eysenck, 1963). Consequently, Neuroticism tends to follow a susceptibility to stress, inefficiency in dealing with frustration, and difficulty controlling impulses (Abbasi, 2016), in this case, in a situation like the COVID-19 pandemic.

#### Agreeableness

Agreeableness, recognized as an important facet of mental health, showed negative associations with Total Difficulties, Conduct Problems, and Hyperactivity/Inattention among the preschoolers in this study. Children with high scores in Agreeableness tend to be cooperative, considerate, empathic, trustworthy, courteous, well-regulated, caring, friendly, and compliant, and exhibit good interpersonal skills (Kochanska and Kim, 2020). Generally, higher scores in Agreeableness in children and adolescents have been associated with improved developmental outcomes, and lower scores in Agreeableness have been associated with multiple symptoms of psychopathology and externalizing and internalizing behavior problems (Laursen et al., 2002, 2010). Studies have also shown a negative association between high scores of Agreeableness and bullying, aggressive and delinquent behaviors, and social problems (Ehrler et al., 1999; Bollmer et al., 2006; Nigg et al., 2020). Previous studies have also shown negative associations between Agreeableness and Hyperactivity and Inattention symptoms (Gomez and Corr, 2014; Nigg et al., 2020).

#### Conscientiousness

Conscientiousness negatively predicted Total difficulty and Hyperactivity among the preschool children. Conscientiousness is characterized by restraining impulses, effortful attention, planned behavior, organization, and goal-oriented behavior (Krieger et al., 2020). In general, individuals who score higher in Conscientious tend to experience less stress and mental health issues when compared to individuals who score lower in Conscientiousness (Wehner et al., 2016). Previous studies have also found a link between low Conscientiousness and Attention-Deficit Hyperactivity Disorder (ADHD) symptoms in some children and adolescents (Cukrowicz et al., 2006; Martel et al., 2009; De Pauw and Mervielde, 2011; Nigg et al., 2020). The inhibitory aspect of Conscientiousness is associated with self-regulation and impulse control which could possibly be indicative of some kind of top-down regulating mechanism (DeYoung, 2010).

#### Extraversion

Extraversion, a trait that has shown strong correlations with mental health outcomes, showed positive associations with Hyperactivity/Inattention and Prosocial Behaviors. Typically, a child with a tendency to Extraversion is likely to be externally focused, and socially active and could be described as outgoing, talkative, assertive, and energetic (Smith et al., 2021). The findings on the associations between Extraversion and Hyperactivity/Inattention have been inconsistent across the literature. Even though some studies have found no significant associations between Hyperactivity/Inattention and Extraversion in children, adolescents, and adults (Martel et al., 2008; De Pauw and Mervielde, 2011; Gomez and Corr, 2014), some have shown strong links between hyperactive–impulsive symptoms and Extraversion (Martel, 2009; Tackett et al., 2012; Gomez and Corr, 2014). Moreover, a study by Gomez and Corr (2014) indicated that positive emotionality (similar to FFM/Extraversion) was associated with inattention, but not with hyperactivity-impulsivity. A possible explanation for the inconsistency of the associations between ADHD symptoms and Extraversion could be that the relationship may be masked when hyperactive, inattention, and impulsive symptoms are not analyzed separately. Moreover, a possible explanation of the positive association between Extraversion and Hyperactivity/Inattention could be related to the COVID-19 situation and the consequences. For instance, children who score higher on Extraversion, usually enjoy social situations, like playing in groups and spending time with their friends but as a result of COVID-19 and the containment measures, they had to spend more time at home, as a result, they might display this suppressed social energy as hyperactivity and inattention symptoms. However, more research is needed to verify this speculation.

Extraversion was the only predictor of Prosocial Behavior in preschoolers. This result is in line with previous studies showing this positive association in children and adolescents (Tariq and Naqvi, 2020; Gómez Tabares and Narvaez Marin, 2022). A possible explanation could be that Extraversion is usually considered a very positive trait (Salmon, 2012), and individuals who score higher on Extraversion usually experience more warmness and positivity (Nguyen et al., 2013), greater social/emotional responsivity (O'connor and Cuevas, 1982), and more positive affect (Morrone et al., 2000), which could be manifested in Prosocial Behavior in children.

### Openness to experience

Openness to Experience did not predict any of the mental health domains in preschoolers.

### 6–18-Year-old children

In 6-18-year-olds, the Total Difficulty score of children was predicted positively by Neuroticism and predicted positively by Extraversion and Conscientiousness. Conduct problems are negatively predicted by Extraversion and Agreeableness. Hyperactivity/Inattention was positively predicted by Extraversion and Neuroticism and negatively predicted by Openness to Experience and Conscientiousness. Peer Relationship Problems are negatively predicted by Extraversion and Prosocial Behavior was positively predicted by Agreeableness. Emotional Symptoms are not predicted by any of the personality traits.

Among the five Coping and Response to COVID-19 Stress factors, Primary Control Coping and Secondary Control Coping are significantly associated with some SDQ subscales (see Table 6). Primary Control Coping is negatively associated with Total Difficulties score, and Secondary Control Coping is negatively associated with Total Difficulties score and Emotional Symptoms. Adaptive coping responses in children include Primary Control Coping (problem-solving, emotional expression, and emotional modulation), and Secondary Control Coping (acceptance, cognitive restructuring, positive thinking, and distraction) (Connor-Smith et al., 2000). Primary Control Coping and Secondary Control Coping are associated with significantly less psychosocial problems, with Secondary Control strategies particularly beneficial for stressful situations that are beyond one’s control (such as COVID-19 situation) (Compas et al., 2017). This is consistent with previous research showing that lower levels of Primary Control Coping and Secondary Control Coping are linked with higher amounts of internalizing symptoms, depression, anxiety, distress, and negative affect in youth (Connor-Smith and Compas, 2004; Evans et al., 2015; Bettis et al., 2016).

### Mental health and personality traits in 6–18-year-old children

#### Extraversion

Extraversion appears to be the strongest predictor of mental health variables in 6-18-year-old children, showing negative associations with Total Difficulty score, Conduct Problems, and Peer Problems and positive associations with Hyperactivity/Inattention. Extraversion is generally linked with higher states of good health (Jokela et al., 2013), as well as mental health (Carver and Scheier, 2014). In the context of the pandemic, this could be explained by the connection found between a higher level of Extraversion and active coping strategies in the form of active problem-solving (Karimzade and Besharat, 2011). Moreover, Extraversion plays a significant role in receiving social support or seeking help during difficult times, such as during the pandemic (Burešová et al., 2020).

There are mixed results regarding the relationship between Extraversion and Conduct Problems. Eysenck and Eysenck (1985) suggested that individuals who score higher on Extraversion are less likely to form conditioned responses than more Introverted individuals, therefore, they are less able to take advantage of aversive conditioning, less sensitive to conditioned stimuli for punishment and are more prone to exhibit antisocial behavior. They also suggested that children with conduct disorder score higher on Extraversion (Eysenck and Eysenck, 1985). More recent studies have also found associations between lower scores of Extroversion with antisocial delinquent behaviors (Krishna, 1993; Komulainen, 2015; Morizot, 2015). However, there are also studies showing no evidence to support Eysenck’s claim that higher scores in Extraversion are associated with delinquent and antisocial behaviors (Fonseca and Yule, 1995; Cale, 2006; Homann, 2019). A possible explanation for the negative association of Conduct Problems and Extraversion in our participants could be related to the COVID-19 situation. For instance, more introverted children who had a few friends at school lost connection with them during the pandemic due to school closures and more Introverted children usually do not reach out to other people (such as siblings and other family members) to fulfill their social needs. It is possible that they manifest their loneliness as aggressive behavior and fighting with others. In contrast, more extroverted children are more likely to reach out to family and friends to satisfy their interpersonal needs in times of school closure. However, more research is needed to test this hypothesis.

As explained in the Study 1 discussion, results are mixed regarding the associations between Extraversion and Hyperactivity/Inattention. The positive relationship between Extraversion and Hyperactivity/Inattention could be explained in the context of the global pandemic, and more Extroverted children and adolescents might manifest their suppressed social energy (as a result of social restriction) as Hyperactivity and Inattention symptoms.

Our results also showed that Extraversion is negatively and significantly associated with Peer Relationship Problems. Our findings are in line with earlier studies finding that adolescents who score higher in Extraversion tend to form and maintain friendships and wider social networks, and to be socially competent (Selfhout et al., 2010). Extraversion is associated with peer acceptance and friendship (Jensen-Campbell et al., 2002), better social interactions (Cheng and Furnham, 2002), sociability, and social interest (Elphick et al., 1998).

#### Conscientiousness

Conscientiousness predicted Total Difficulty and Hyperactivity/Inattention in 6-18-year-olds, as well as in preschoolers. This is in line with previous studies showing that Conscientiousness is associated with Inattention in children (Martel et al., 2008, 2009) and Hyperactivity-Impulsivity in adolescents (Martel et al., 2009). As explained in Study 1 discussion, a possible explanation could be that Hyperactivity/Inattention could be associated with executive control and Conscientiousness, indicative of underlying top-down regulatory processes (Nigg, 2010).

#### Neuroticism

Neuroticism is positively associated with Total Difficulty score and Hyperactivity/Inattention in 6-18-year-olds. Overall, Neuroticism is directly related to psychopathology, and individuals who score higher in Neuroticism are more likely to develop Axis I psychopathology, particularly the common mental disorders including mood, anxiety, substance use disorders, and also schizophrenia, bipolar disorder, and ADHD (Gale et al., 2016). Our results are consistent with previous studies showing associations between Neuroticism and Hyperactivity/Inattention (Martel et al., 2010; Krieger et al., 2020). High Neuroticism has also been linked with the persistence of hyperactivity and inattention during adolescence (Miller et al., 2008).

#### Agreeableness

Agreeableness showed negative associations with Conduct Problems and Positive associations with Prosocial Behaviors. The concept of Prosocial Behavior overlaps substantially with the construct of Agreeableness and it is even sometimes considered a form of Agreeableness (Graziano and Eisenberg, 1997). Prosocial tendencies contribute to responsible and helpful behavior, constructs defining Agreeableness (Caspi et al., 2005). Agreeableness has been consistently associated with Prosocial Behaviors during childhood (Graziano et al., 1997) and adolescence (Shiner, 2000). Our results regarding the negative associations of Agreeableness with Conduct Problems are in line with previous studies (Ehrler et al., 1999; Bollmer et al., 2006; Nigg et al., 2020). Some studies have also shown that Agreeableness in childhood could predict Aggressive behavior and Conduct symptoms in adolescence (Shiner, 2000; Gleason et al., 2004).

#### Openness to experience

Openness to Experience is negatively and significantly associated with Hyperactivity/Inattention in 6-18-year-olds. Openness to Experience refers to the degree to which an individual actively seeks out new experiences and accepts and explores new situations (Pervin, 2003). Generally, individuals who score higher on Openness to Experience are more likely to experience higher psychological well-being (Jacobsson et al., 2021). There are some studies in adults showing a negative relationship between Openness to Experience and Hyperactivity/Inattention (Smith and Martel, 2019; Blanken et al., 2021), and some showing no associations (Krieger et al., 2020; Nigg et al., 2020). We only found one study on 8–12-year-old children, showing that children with Hyperactivity/Inattention symptoms were consistently rated as having lower Openness to Experience (Casher, 2016). A possible explanation for this negative association is that Openness to Experience is generally related to higher performance of children in school and on cognitive tests and is also related to some elements of intellect (Nave et al., 2017).

## General conclusion

As of today, more than 6.6 million people have died from the coronavirus COVID-19 outbreak (Worldometers, 2022) and the global COVID-19 pandemic and the consequent economic recession and social restrictions have adversely affected the mental health of many people including children. Studies have reported various mental health problems among children and adolescents exposed to the COVID-19 pandemic, including anxiety, stress, depression, panic, irritation, impulsivity, loneliness, fatigue, and confusion (Hossain et al., 2020; Jiao et al., 2020; Theberath et al., 2022).

There are some studies showing the contribution of personality traits to well-being of adults during the pandemic (Shokrkon and Nicoladis, 2021; Lo et al., 2022; Odachi et al., 2022), however, our study seems to be the first study investigating this relationship in children. The results of our two studies showed that personality traits in children and adolescents contribute to their mental health status during the pandemic. In preschoolers, Neuroticism and Agreeableness predicted the most Difficulty subscales of SDQ, and in 6-18-year-old children, Extraversion predicted the most Difficulty subscales of SDQ. Also, Openness to Experience was the weakest predictor of mental health status in Canadian youth. Moreover, in preschoolers among the mental health subscales, the Total Difficulty score and Hyperactivity/Inattention seem to have the strongest associations with personality traits and Peer Relationship Problems have the weakest associations. In 6-18-year-olds, Total Difficulty scores and Conduct Problems are most strongly associated with personality traits, and Hyperactivity/Inattention is least strongly associated with personality traits.

In comparing the results of preschoolers and 6–18-year-old children, we can observe different patterns. Specifically, in 6–18-year-old children, Extraversion is associated negatively with 3 Difficulty subscales of SDQ, however, this association is not observed in preschool children. A possible explanation could be related to the experience of schooling that older children had. For example, it could be that more Extraverted school-aged children found more friends at school and maintained their friendships during the pandemic using the social media, as a result they were able to better maintain their positive mental health compared to more Extraverted preschoolers who did not have the experience of schooling.

There are some limitations to the current study that should be considered. Despite aiming for participants from all over Canada, the majority of our sample resided in Alberta (the province where the study was conducted). The second limitation of this study is that our data were collected only at one point in time during the second year of COVID-19, and since it was summer, people were more likely to spend time outdoors which could affect the results of our study. These limitations could limit the generalizability of our results.

Regardless of the mentioned limitations, our study has important implications, as it is necessary to understand how personality traits contribute to the mental health and well-being of children in order to provide them with mental health care that is tailored to their personality traits. The results of our study could help public health services provide mental health services that are personality-appropriate during and after this pandemic. More individually appropriate child and adolescent mental health treatment at all phases of the pandemic is an unmet urgent need for long-term mental health impacts of children and adolescents.

## Data availability statement

The datasets presented in this study can be found in online repositories. The names of the repository/repositories and accession number(s) can be found below: https://figshare.com/articles/dataset/PRESCHOOL_covid_sav/22305910.

## Ethics statement

The studies involving human participants were reviewed and approved by the Research Ethics Board at the University of Alberta (Pro00100751). The patients/participants provided their written informed consent to participate in this study.

## Author contributions

AS was responsible for the conceptualization, data collection, and analysis as well as the manuscript composition. EN was responsible for the supervision, reviewing, and editing. All authors contributed to the article and approved the submitted version.

## Funding

This study received funding from a Discovery Grant (#2018–04978) from the Natural Sciences and Engineering Research Council of Canada to the EN.

## Conflict of interest

The authors declare that the research was conducted in the absence of any commercial or financial relationships that could be construed as a potential conflict of interest.

## Publisher’s note

All claims expressed in this article are solely those of the authors and do not necessarily represent those of their affiliated organizations, or those of the publisher, the editors and the reviewers. Any product that may be evaluated in this article, or claim that may be made by its manufacturer, is not guaranteed or endorsed by the publisher.

## References

[ref1] Abbasi I. S. (2016). The role of neuroticism in the maintenance of chronic baseline stress perception and negative affect. Span. J. Psychol. 19:E9. doi: 10.1017/sjp.2016.7, PMID: 26940336

[ref2] Abe J. A. A. Izard C. E. (1999). A longitudinal study of emotion expression and personality relations in early development. J. Pers. Soc. Psychol. 77, 566–577. doi: 10.1037/0022-3514.77.3.566 10510509

[ref3] Agh-Yousefi A. Maleki B. (2011). Prediction of depression symptoms by personality traits in children. J. Clin. Psychol. 3, 9–17.

[ref4] Albuquerque I. de Lima M. P. Matos M. Figueiredo C. (2012). Personality and subjective well-being: what hides behind global analyses? Soc. Indic. Res. 105, 447–460. doi: 10.1007/s11205-010-9780-7

[ref5] Ali P. Rogers M. Heward-Belle S. (2021). COVID-19 and domestic violence: impact on mental health. J. Crim. Psychol. 11, 188–202. doi: 10.1108/JCP-12-2020-0050

[ref6] Andrés M. L. Richaud de Minzi M. C. Castañeiras C. Canet-Juric L. Rodríguez-Carvajal R. (2016). Neuroticism and depression in children: the role of cognitive emotion regulation strategies. J. Genet. Psychol. 177, 55–71. doi: 10.1080/00221325.2016.1148659, PMID: 27010452

[ref7] Araújo L. A. D. Veloso C. F. Souza M. D. C. Azevedo J. M. C. D. Tarro G. (2021). The potential impact of the COVID-19 pandemic on child growth and development: a systematic review. J. Pediatr. 97, 369–377. doi: 10.1016/j.jped.2020.08.008, PMID: 32980318 PMC7510529

[ref8] Asendorpf J. B. Denissen J. J. (2006). Predictive validity of personality types versus personality dimensions from early childhood to adulthood: implications for the distinction between core and surface traits. Merrill-Palmer Quar. 52, 486–513.

[ref9] Barbaranelli C. Caprara G. V. Rabasca A. Pastorelli C. (2003). A questionnaire for measuring the big five in late childhood. Personal. Individ. Differ. 34, 645–664. doi: 10.1016/S0191-8869(02)00051-X

[ref10] Bettis A. H. Forehand R. McKee L. Dunbar J. P. Watson K. H. Compas B. E. (2016). Testing specificity: associations of stress and coping with symptoms of anxiety and depression in youth. J. Child Fam. Stud. 25, 949–958. doi: 10.1007/s10826-015-0270-z, PMID: 28794609 PMC5546749

[ref11] Biggs A. Brough P. Drummond S. (2017). “Lazarus and Folkman’s psychological stress and coping theory” in The handbook of stress and health: a guide to research and practice. eds. C. L. Cooper and J. C. Quick (Wiley Blackwell), 351–364.

[ref12] Blanken T. F. Courbet O. Franc N. Albajara Sáenz A. Van Someren E. J. Peigneux P.. (2021). Is an irritable ADHD profile traceable using personality dimensions? Replicability, stability, and predictive value over time of data-driven profiles. Eur. Child Adolesc. Psychiatry 30, 633–645. doi: 10.1007/s00787-020-01546-z, PMID: 32399809 PMC8041702

[ref13] Blöte A. W. Miers A. C. Westenberg P. M. (2022). Concurrent and prospective associations between social anxiety and responses to stress in adolescence. Res. Child Adolesc. Psychopathol. 50, 659–668. doi: 10.1007/s10802-021-00880-3, PMID: 34661781 PMC9054901

[ref14] Bollmer J. M. Harris M. J. Milich R. (2006). Reactions to bullying and peer victimization: narratives, physiological arousal, and personality. J. Res. Pers. 40, 803–828. doi: 10.1016/j.jrp.2005.09.003

[ref15] Boyce W. T. Chesney M. Alkon A. Tschann J. M. Adams S. Chesterman B.. (1995). Psychobiologic reactivity to stress and childhood respiratory illnesses: results of two prospective studies. Psychosom. Med. 57, 411–422. doi: 10.1097/00006842-199509000-00001, PMID: 8552730

[ref16] Broeren S. Muris P. Bouwmeester S. van der Heijden K. B. Abee A. (2011). The role of repetitive negative thoughts in the vulnerability for emotional problems in non-clinical children. J. Child Fam. Stud. 20, 135–148. doi: 10.1007/s10826-010-9380-9, PMID: 21475413 PMC3048292

[ref17] Bucher M. A. Suzuki T. Samuel D. B. (2019). A meta-analytic review of personality traits and their associations with mental health treatment outcomes. Clin. Psychol. Rev. 70, 51–63. doi: 10.1016/j.cpr.2019.04.002, PMID: 30981042

[ref18] Buheji M. Hassani A. Ebrahim A. Costa Cunha K. Jahrami H. Baloshi M.. (2020). Children and coping during COVID-19: a scoping review of bio-psycho-social factors. Int. J. Appl. Psychol. 10, 8–15. doi: 10.5923/j.Ijap.20201001.02

[ref19] Burešová I. Jelínek M. Dosedlová J. Klimusová H. (2020). Predictors of mental health in adolescence: the role of personality, dispositional optimism, and social support. SAGE Open 10:215824402091796. doi: 10.1177/2158244020917963

[ref20] Cale E. M. (2006). A quantitative review of the relations between the “big 3” higher order personality dimensions and antisocial behavior. J. Res. Pers. 40, 250–284. doi: 10.1016/j.jrp.2005.01.001

[ref01] Carver C. S. Scheier M. F. (2014). Dispositional optimism. Trends Cogni. Sci. 18, 293–299. 10.1016/j.tics.2014.02.003 PMC4061570 24630971

[ref21] Carver C. S. Scheier M. F. Weintraub J. K. (1989). Assessing coping strategies: a theoretically based approach. J. Pers. Soc. Psychol. 56, 267–283. doi: 10.1037/0022-3514.56.2.267 2926629

[ref22] Casher G. A. (2016). The big five and ADHD: an investigation of subtypes and emotional regulation. Carbondale: Southern Illinois University.

[ref23] Caspi A. Roberts B. W. Shiner R. L. (2005). Personality development: stability and change. Annu. Rev. Psychol. 56, 453–484. doi: 10.1146/annurev.psych.55.090902.141913 15709943

[ref24] Cheng H. Furnham A. (2002). Personality, peer relations, and self-confidence as predictors of happiness and loneliness. J. Adolesc. 25, 327–339. doi: 10.1006/jado.2002.0475, PMID: 12128043

[ref25] Coiro M. J. Watson K. H. Ciriegio A. Jones M. Wolfson A. R. Reisman J.. (2021). Coping with COVID-19 stress: associations with depression and anxiety in a diverse sample of US adults. Curr. Psychol. 1–13. doi: 10.1007/s12144-021-02444-6, PMID: 34754165 PMC8568066

[ref26] Compas B. E. Connor J. Osowiecki D. Welch A. (1997). “Effortful and involuntary responses to stress: Implications for coping with chronic stres,” in Coping with chronic stress. ed. B. H. Gottlieb (Plenum Press), 105–130.

[ref27] Compas B. E. Jaser S. S. Bettis A. H. Watson K. H. Gruhn M. Dunbar J. P.. (2017). Coping, emotion regulation, and psychopathology in childhood and adolescence: a meta-analytic and narrative review. Psychol. Bull. 143, 939–991. doi: 10.1037/bul0000110, PMID: 28616996 PMC7310319

[ref28] Connor-Smith J. K. Compas B. E. (2004). Coping as a moderator of relations between reactivity to interpersonal stress, health status, and internalizing problems. Cogn. Ther. Res. 28, 347–368. doi: 10.1023/B:COTR.0000031806.25021.d5

[ref29] Connor-Smith J. K. Compas B. E. Wadsworth M. E. Thomsen A. H. Saltzman H. (2000). Responses to stress in adolescence: measurement of coping and involuntary stress responses. J. Consult. Clin. Psychol. 68, 976–992. doi: 10.1037/0022-006X.68.6.976, PMID: 11142550

[ref30] Corbett B. A. Muscatello R. A. Klemencic M. E. Schwartzman J. M. (2021). The impact of COVID-19 on stress, anxiety, and coping in youth with and without autism and their parents. Autism Res. 14, 1496–1511. doi: 10.1002/aur.2521, PMID: 33913261 PMC8237027

[ref31] Costello E. J. Compton S. N. Keeler G. Angold A. (2003). Relationships between poverty and psychopathology: a natural experiment. JAMA 290, 2023–2029. doi: 10.1001/jama.290.15.2023 14559956

[ref32] Croft S. Stride C. Maughan B. Rowe R. (2015). Validity of the strengths and difficulties questionnaire in preschool-aged children. Pediatrics 135, e1210–e1219. doi: 10.1542/peds.2014-2920, PMID: 25847804

[ref33] Cukrowicz K. C. Taylor J. Schatschneider C. Iacono W. G. (2006). Personality differences in children and adolescents with attention-deficit/hyperactivity disorder, conduct disorder, and controls. J. Child Psychol. Psychiatry 47, 151–159. doi: 10.1111/j.1469-7610.2005.01461.x 16423146

[ref34] Davies S. Aurini J. (2021). Estimate of student learning losses from Covid-19 school closures. Ottawa: Royal Society of Canada.

[ref35] De Pauw S. S. Mervielde I. (2011). The role of temperament and personality in problem behaviors of children with ADHD. J. Abnorm. Child Psychol. 39, 277–291. doi: 10.1007/s10802-010-9459-1, PMID: 20862537

[ref36] Deoni S. C. Beauchemin J. Volpe A. D’Sa V. RESONANCE Consortium. (2022). The COVID-19 pandemic and early child cognitive development: a comparison of development in children born during the pandemic and historical references. medRxiv, 2021-08.

[ref37] DeYoung C. G. (2010). Personality neuroscience and the biology of traits. Soc. Personal. Psychol. Compass 4, 1165–1180. doi: 10.1111/j.1751-9004.2010.00327.x

[ref38] Dufton L. M. Dunn M. J. Slosky L. S. Compas B. E. (2010). Self-reported and laboratory-based responses to stress in children with recurrent pain and anxiety. J. Pediatr. Psychol. 36, 95–105. doi: 10.1093/jpepsy/jsq070, PMID: 20736388 PMC3021808

[ref39] Egan S. M. Pope J. Moloney M. Hoyne C. Beatty C. (2021). Missing early education and care during the pandemic: the socio-emotional impact of the COVID-19 crisis on young children. Early Childhood Educ. J. 49, 925–934. doi: 10.1007/s10643-021-01193-2, PMID: 33935481 PMC8076664

[ref40] Ehrler D. J. Evans J. G. McGhee R. L. (1999). Extending big-five theory into childhood: a preliminary investigation into the relationship between big-five personality traits and behavior problems in children. Psychol. Sch. 36, 451–458. doi: 10.1002/(SICI)1520-6807(199911)36:6<451::AID-PITS1>3.0.CO;2-E

[ref02] Elphick E. Halverson C. F. Marzal-Wisniewska M. (1998). Extraversion: Toward a unifying description from infancy to adulthood. Parental Descript. Child Person. Develop. Antecedents Big Five, 21–48.

[ref41] Evans L. D. Kouros C. Frankel S. A. McCauley E. Diamond G. S. Schloredt K. A.. (2015). Longitudinal relations between stress and depressive symptoms in youth: coping as a mediator. J. Abnorm. Child Psychol. 43, 355–368. doi: 10.1007/s10802-014-9906-5, PMID: 24993312 PMC4284153

[ref42] Eysenck H. J. (1963). Biological basis of personality. Nature 199, 1031–1034. doi: 10.1038/1991031a0 14066934

[ref43] Eysenck H. J. (1992). Four ways five factors are not basic. Personal. Individ. Differ. 13, 667–673. doi: 10.1016/0191-8869(92)90237-J

[ref05] Eysenck H. J. Eysenck M. W. (1985). Personality and Individual Differences: A Natural Science Approach. Plenum, New York.

[ref44] Fonseca A. C. Yule W. (1995). Personality and antisocial behavior in children and adolescents: an enquiry into Eysenck's and Gray's theories. J. Abnorm. Child Psychol. 23, 767–781. doi: 10.1007/BF01447476, PMID: 8609312

[ref45] Gadermann A. C. Thomson K. C. Richardson C. G. Gagné M. McAuliffe C. Hirani S.. (2021). Examining the impacts of the COVID-19 pandemic on family mental health in Canada: findings from a national cross-sectional study. BMJ Open 11:e042871. doi: 10.1136/bmjopen-2020-042871, PMID: 33436472 PMC7804831

[ref46] Gale C. R. Hagenaars S. P. Davies G. Hill W. D. Liewald D. C. Cullen B.. (2016). Pleiotropy between neuroticism and physical and mental health: findings from 108 038 men and women in UK biobank. Transl. Psychiatry 6:e791. doi: 10.1038/tp.2016.56, PMID: 27115122 PMC4872414

[ref47] Gleason K. A. Jensen-Campbell L. A. South Richardson D. (2004). Agreeableness as a predictor of aggression in adolescence. Aggress. Behav. Off. J. Int. Soc. Res. Aggress. 30, 43–61. doi: 10.1002/ab.20002

[ref48] Goldberg L. R. (1992). The development of markers for the big-five factor structure. Psychol. Assess. 4, 26–42. doi: 10.1037/1040-3590.4.1.26

[ref49] Goldberg L. R. (1993). The structure of phenotypic personality traits. Am. Psychol. 48, 26–34. doi: 10.1037/0003-066X.48.1.26 8427480

[ref50] Gomez R. Corr P. J. (2014). ADHD and personality: a meta-analytic review. Clin. Psychol. Rev. 34, 376–388. doi: 10.1016/j.cpr.2014.05.002 24929793

[ref51] Gómez Tabares A. S. Narvaez Marin M. (2022). Dimensiones de la personalidad y su relación con las tendencias prosociales y la empatía en niños (as) y adolescentes en vulnerabilidad psicosocial. Revista de Psicología (PUCP) 40, 37–72. doi: 10.18800/psico.202201.002

[ref52] Goodman R. (1997). The strengths and difficulties questionnaire: a research note. J. Child Psychol. Psychiatry 38, 581–586. doi: 10.1111/j.1469-7610.1997.tb01545.x 9255702

[ref53] Graziano W. G. Eisenberg N. (1997). “Agreeableness: a dimension of personality” in Handbook of personality psychology. eds. R. Hogan, J. A. Johnson and S. R. Briggs (Cambridge, MA: Academic Press).

[ref54] Graziano W. G. Jensen-Campbell L. A. Finch J. F. (1997). The self as a mediator between personality and adjustment. J. Pers. Soc. Psychol. 73, 392–404. doi: 10.1037/0022-3514.73.2.392 9248056

[ref55] Grist C. L. McCord D. M. (2010). Individual differences in preschool children: temperament or personality? Infant Child Dev. Int. J. Res. Pract. 19, n/a–274. doi: 10.1002/icd.663

[ref56] Grist C. L. Socha A. McCord D. M. (2012). The M5–PS–35: a five-factor personality questionnaire for preschool children. J. Pers. Assess. 94, 287–295. doi: 10.1080/00223891.2011.653063, PMID: 22309713

[ref57] Halverson C. F. Havill V. L. Deal J. Baker S. R. Victor J. B. Pavlopoulos V.. (2003). Personality structure as derived from parental ratings of free descriptions of children: the inventory of child individual differences. J. Pers. 71, 995–1026. doi: 10.1111/1467-6494.7106005, PMID: 14633056

[ref58] Hoffmann M. D. Lang J. J. Guerrero M. D. Cameron J. D. Goldfield G. S. Orpana H. M.. (2020). Evaluating the psychometric properties of the parent-rated strengths and difficulties questionnaire in a nationally representative sample of Canadian children and adolescents aged 6 to 17 years. Health Rep. 31, 13–20. doi: 10.25318/82-003-x202000800002-eng, PMID: 32816414

[ref59] Homann J. (2019). An investigation into the role of risk opportunity in the relationship between delinquency and extraversion Bachelor's thesis University of Twente.

[ref60] Hossain M. M. Tasnim S. Sultana A. Faizah F. Mazumder H. Zou L.. (2020). Epidemiology of mental health problems in COVID-19: a review. F1000Res 9:636. doi: 10.12688/f1000research.24457.1, PMID: 33093946 PMC7549174

[ref61] Idoiaga N. Berasategi N. Eiguren A. Picaza M. (2020). Exploring children’s social and emotional representations of the COVID-19 pandemic. Front. Psychol. 11:1952. doi: 10.3389/fpsyg.2020.01952, PMID: 32922334 PMC7456920

[ref62] Jacobsson P. Hopwood C. J. Söderpalm B. Nilsson T. (2021). Adult ADHD and emerging models of maladaptive personality: a meta-analytic review. BMC Psychiatry 21, 1–16. doi: 10.1186/s12888-021-03284-1 34074265 PMC8170979

[ref63] Jensen-Campbell L. A. Adams R. Perry D. G. Workman K. A. Furdella J. Q. Egan S. K. (2002). Agreeableness, extraversion, and peer relations in early adolescence: winning friends and deflecting aggression. J. Res. Pers. 36, 224–251. doi: 10.1006/jrpe.2002.2348

[ref64] Jiao W. Y. Wang L. N. Liu J. Fang S. F. Jiao F. Y. Pettoello-Mantovani M.. (2020). Behavioral and emotional disorders in children during the COVID-19 epidemic. J. Pediatr. 221, 264–266.e1. doi: 10.1016/j.jpeds.2020.03.013, PMID: 32248989 PMC7127630

[ref65] John O. P. Naumann L. P. Soto C. J. (2008). Paradigm shift to the integrative big five trait taxonomy: History, measurement, and conceptual issues. New York: The Guilford Press.

[ref03] Jokela M. Batty G. D. Nyberg S. T. Virtanen M. Nabi H. Singh-Manoux A.. (2013). Personality and all-cause mortality: individual-participant meta-analysis of 3,947 deaths in 76,150 adults. Am. J. Epidemiol. 178, 667–675. 23911610 10.1093/aje/kwt170 PMC3755650

[ref66] Kang S. Sun Y. Zhang X. Sun F. Wang B. Zhu W. (2021). Is physical activity associated with mental health among Chinese adolescents during isolation in COVID-19 pandemic? J. Epidemiol. Global Health 11:26. doi: 10.2991/jegh.k.200908.001 PMC7958283 32959611

[ref04] Karimzade A. Besharat M. A. (2011). An investigation of the relationship between personality dimensions and stress coping styles. Procedia-Social Behav. Sci. 30, 797–802.

[ref69] Kochanska G. Kim S. (2020). Children’s early difficulty and agreeableness in adolescence: testing a developmental model of interplay of parent and child effects. Dev. Psychol. 56, 1556–1564. doi: 10.1037/dev0001023, PMID: 32510231 PMC8262374

[ref70] Komulainen K. (2015). Association of personality with adolescent delinquency in a context of deviant socialization: Persoonallisuuden yhteys nuorisorikollisuuteen rikollisen sosialisaation kontekstissa Doctoral dissertation University of Helsinki.

[ref06] Krieger V. Amador-Campos J. A. Guàrdia-Olmos J. (2020). Executive functions, Personality traits and ADHD symptoms in adolescents: A mediation analysis. PloS One 15:e0232470. 32374779 10.1371/journal.pone.0232470 PMC7202790

[ref71] Krishna U. (1993). Adolescent's delinquent behavior and personality. Indian J. Criminol. 21, 90–94.

[ref72] Kumar A. Nayar K. R. (2021). COVID 19 and its mental health consequences. J. Ment. Health 30, 1–2. doi: 10.1080/09638237.2020.1757052 32339041

[ref07] Lahey B. B. (2009). Public health significance of neuroticism. Am. Psychol. 64:241. 19449983 10.1037/a0015309 PMC2792076

[ref73] Laursen B. Hafen C. A. Rubin K. H. Booth-LaForce C. Rose-Krasnor L. (2010). The distinctive difficulties of disagreeable youth. Merrill-Palmer Quarterly 56, 80–103. doi: 10.1353/mpq.0.0040, PMID: 35812133 PMC9269991

[ref74] Laursen B. Pulkkinen L. Adams R. (2002). The antecedents and correlates of agreeableness in adulthood. Dev. Psychol. 38, 591–603. doi: 10.1037/0012-1649.38.4.591, PMID: 12090488 PMC2730208

[ref75] Lo C. F. Leung F. K. Y. Lui C. P. F. Ng E. C. B. (2022). Predictive effect of extraversion and neuroticism on mental health during the Covid-19 pandemic in Hong Kong: the mediating role of coping strategies. Psychology 13, 1391–1412. doi: 10.4236/psych.2022.139089

[ref76] Martel M. M. (2009). Research review: a new perspective on attention-deficit/hyperactivity disorder: emotion dysregulation and trait models. J. Child Psychol. Psychiatry 50, 1042–1051. doi: 10.1111/j.1469-7610.2009.02105.x, PMID: 19508495

[ref77] Martel M. M. Nigg J. T. Lucas R. E. (2008). Trait mechanisms in youth with and without attention-deficit/hyperactivity disorder. J. Res. Pers. 42, 895–913. doi: 10.1016/j.jrp.2007.12.004, PMID: 19649133 PMC2597829

[ref78] Martel M. M. Nigg J. T. Von Eye A. (2009). How do trait dimensions map onto ADHD symptom domains? J. Abnorm. Child Psychol. 37, 337–348. doi: 10.1007/s10802-008-9255-3, PMID: 18668361 PMC2818785

[ref79] Martel M. M. Nikolas M. Jernigan K. Friderici K. Nigg J. T. (2010). Personality mediation of genetic effects on attention-deficit/hyperactivity disorder. J. Abnorm. Child Psychol. 38, 633–643. doi: 10.1007/s10802-010-9392-3, PMID: 20146095 PMC4303410

[ref80] Matthews K. A. Hall M. H. Cousins J. Lee L. (2016). Getting a good night's sleep in adolescence: do strategies for coping with stress matter? Behav. Sleep Med. 14, 367–377. doi: 10.1080/15402002.2015.1007994, PMID: 26371884 PMC4792790

[ref81] McCrae R. R. Costa P. T. (1987). Validation of the five-factor model of personality across instruments and observers. J. Pers. Soc. Psychol. 52, 81–90. doi: 10.1037/0022-3514.52.1.81, PMID: 3820081

[ref82] McNamara L. (2021). Redesigning the recess experience: lessons from COVID-19. Ottawa: Royal Society of Canada.

[ref83] Miller C. J. Miller S. R. Newcorn J. H. Halperin J. M. (2008). Personality characteristics associated with persistent ADHD in late adolescence. J. Abnorm. Child Psychol. 36, 165–173. doi: 10.1007/s10802-007-9167-7, PMID: 17701339

[ref84] Moore S. A. Faulkner G. Rhodes R. E. Brussoni M. Chulak-Bozzer T. Ferguson L. J.. (2020). Impact of the COVID-19 virus outbreak on movement and play behaviours of Canadian children and youth: a national survey. Int. J. Behav. Nutr. Phys. Act. 17, 1–11. doi: 10.1186/s12966-020-00987-8 32631350 PMC7336091

[ref85] Moos R. H. Holahan C. J. (2003). Dispositional and contextual perspectives on coping: toward an integrative framework. J. Clin. Psychol. 59, 1387–1403. doi: 10.1002/jclp.10229, PMID: 14618606

[ref86] Morizot J. (2015). The contribution of temperament and personality traits to criminal and antisocial behavior development and desistance. Dev. Crim. Antisoc. Behav., 137–165. doi: 10.1007/978-3-319-08720-7_10

[ref87] Morrone J. V. Depue R. A. Scherer A. J. White T. L. (2000). Film-induced incentive motivation and positive activation in relation to agentic and affiliative components of extraversion. Personal. Individ. Differ. 29, 199–216. doi: 10.1016/S0191-8869(99)00187-7

[ref88] Nave C. S. Edmonds G. W. Hampson S. E. Murzyn T. Sauerberger K. S. (2017). From elementary school to midlife: childhood personality predicts behavior during cognitive testing over four decades later. J. Res. Pers. 67, 183–189. doi: 10.1016/j.jrp.2016.10.001, PMID: 28579657 PMC5451157

[ref89] Nguyen H. V. Koo K. H. Granato H. F. George W. H. (2013). Interventions for addiction: Chapter 96. Understanding individual variation in student alcohol use. Amsterdam: Elsevier Inc. Chapters.

[ref90] Nigg J. T. (2010). Attention-deficit/hyperactivity disorder: Endophenotypes, structure, and etiological pathways. Curr. Dir. Psychol. Sci. 19, 24–29. doi: 10.1177/0963721409359282

[ref91] Nigg J. T. John O. P. Blaskey L. G. Huang-Pollock C. L. Willcutt E. G. Hinshaw S. P.. (2020). Pediatric COVID-19: systematic review of the literature. Am. J. Otolaryngol. 41:102573. doi: 10.1016/j.amjoto.2020.102573, PMID: 32531620 PMC7833675

[ref92] O'connor M. Cuevas J. (1982). The relationship of children's prosocial behavior to social responsibility, prosocial reasoning, and personality. J. Genet. Psychol. 140, 33–45. doi: 10.1080/00221325.1982.10534173

[ref93] Odachi R. Takahashi S. Sugawara D. Tabata M. Kajiwara T. Hironishi M.. (2022). The big five personality traits and the fear of COVID-19 in predicting depression and anxiety among Japanese nurses caring for COVID-19 patients: a cross-sectional study in Wakayama prefecture. PLoS One 17:e0276803. doi: 10.1371/journal.pone.0276803, PMID: 36301905 PMC9612447

[ref95] Pervin L. A. (2003). The science of personality. Oxford: Oxford University Press.

[ref96] Proto E. Zhang A. (2021). COVID-19 and mental health of individuals with different personalities. Proc. Natl. Acad. Sci. U. S.A. 118:e2109282118. doi: 10.1073/pnas.2109282118 34508007 PMC8449367

[ref97] Ramaswamy S. Seshadri S. (2020). Children on the brink: risks for child protection, sexual abuse, and related mental health problems in the COVID-19 pandemic. Indian J. Psychiatry 62, S404–S413. doi: 10.4103/psychiatry.IndianJPsychiatry_1032_20, PMID: 33227060 PMC7659798

[ref98] Rosenberg H. J. Jankowski M. K. Fortuna L. R. Rosenberg S. D. Mueser K. T. (2011). A pilot study of a cognitive restructuring program for treating posttraumatic disorders in adolescents. Psychol. Trauma Theory Res. Pract. Policy 3, 94–99. doi: 10.1037/a0019889

[ref99] Rothbart M. K. Ahadi S. A. Evans D. E. (2000). Temperament and personality: origins and outcomes. J. Pers. Soc. Psychol. 78, 122–135. doi: 10.1037/0022-3514.78.1.122 10653510

[ref100] Salmon C. (2012). Birth order, effect on personality, and behavior. Encycl. Hum. Behav., 353–359. doi: 10.1016/B978-0-12-375000-6.00064-1

[ref101] Sauer-Zavala S. Wilner J. G. Barlow D. H. (2017). Addressing neuroticism in psychological treatment. Personal. Disord. Theory Res. Treat. 8, 191–198. doi: 10.1037/per0000224 29120218

[ref102] Selfhout M. Burk W. Branje S. Denissen J. Van Aken M. Meeus W. (2010). Emerging late adolescent friendship networks and big five personality traits: a social network approach. J. Pers. 78, 509–538. doi: 10.1111/j.1467-6494.2010.00625.x, PMID: 20433629

[ref103] Sexton K. A. Norton P. J. Walker J. R. Norton G. R. (2003). Hierarchical model of generalized and specific vulnerabilities in anxiety. Cogn. Behav. Ther. 32, 82–94. doi: 10.1080/16506070302321 16291539

[ref104] She J. Liu L. Liu W. (2020). COVID-19 epidemic: disease characteristics in children. J. Med. Virol. 92, 747–754. doi: 10.1002/jmv.25807, PMID: 32232980 PMC7228385

[ref105] Shiner R. L. (2000). Linking childhood personality with adaptation: evidence for continuity and change across time into late adolescence. J. Pers. Soc. Psychol. 78, 310–325. doi: 10.1037/0022-3514.78.2.310, PMID: 10707337

[ref106] Shokrkon A. Nicoladis E. (2021). How personality traits of neuroticism and extroversion predict the effects of the COVID-19 on the mental health of Canadians. PLoS One 16:e0251097. doi: 10.1371/journal.pone.0251097, PMID: 34010299 PMC8133466

[ref107] Singer A. J. Glenwick D. S. Danyko S. (2000). Stress responses of adolescents in residential treatment: a research note. Resid. Treat. Child. Youth 17, 67–82. doi: 10.1300/J007v17n04_06

[ref108] Smith J. Guimond F. A. Bergeron J. St-Amand J. Fitzpatrick C. Gagnon M. (2021). Changes in students’ achievement motivation in the context of the COVID-19 pandemic: a function of extraversion/introversion? Educ. Sci. 11:30. doi: 10.3390/educsci11010030

[ref109] Smith T. E. Martel M. M. (2019). Trait-based profiles of ADHD in adolescents and young adults. J. Clin. Child Adolesc. Psychol. 48, 440–454. doi: 10.1080/15374416.2018.1491004, PMID: 30028226

[ref110] Sprang G. Silman M. (2013). Posttraumatic stress disorder in parents and youth after health-related disasters. Disaster Med. Public Health Prep. 7, 105–110. doi: 10.1017/dmp.2013.22, PMID: 24618142

[ref111] Statistic Canada (2021). Survey on COVID-19 and mental health, February to May 2021. Available at: https://www150.statcan.gc.ca/n1/daily-quotidien/210927/dq210927a-eng.htm (Accessed April 5, 2023).

[ref113] Strickhouser J. E. Zell E. Krizan Z. (2017). Does personality predict health and well-being? A metasynthesis. Health Psychol. 36, 797–810. doi: 10.1037/hea0000475, PMID: 28277701

[ref114] Tackett J. L. Martel M. M. Kushner S. C. (2012). Temperament, externalizing disorders, and attention-deficit/hyperactivity disorder. Psych. Investig. 16, 206–212. doi: 10.30773/pi.2019.01.10.1

[ref115] Tariq F. T. Naqvi I. (2020). Relationship between personality traits and prosocial behavior among adolescents. Found. Univ. J. Psychol. 4, 54–63.

[ref116] Theberath M. Bauer D. Chen W. Salinas M. Mohabbat A. B. Yang J.. (2022). Effects of COVID-19 pandemic on mental health of children and adolescents: a systematic review of survey studies. SAGE Open Med. 10:210867. doi: 10.1177/20503121221086712 PMC8972920 35371484

[ref117] Vaillancourt T. Szatmari P. Georgiades K. Krygsman A. (2021). The impact of COVID-19 on the mental health of Canadian children and youth. Facets 6, 1628–1648. doi: 10.1139/facets-2021-0078

[ref118] van Berkel H. K. (2009). The relationship between personality, coping styles and stress, anxiety and depression A thesis submitted in partial fulfilment of the requirements for the Degree of Master of Science in Psychology.

[ref08] Vreeke L. J. Muris P. (2012). Relations between behavioral inhibition, Big Five personality factors, and anxiety disorder symptoms in non-clinical and clinically anxious children. Child Psychiatry Hum. Develop. 43, 884–894. doi: 10.1007/s10578-012-0302-5 PMC3472051 22528030

[ref119] Wehner C. Schils T. Borghans L. (2016). Personality and mental health: the role and substitution effect of emotional stability and conscientiousness. IZA Discussion Papers. doi: 10.2139/ssrn.2864838,

[ref120] Wolff B. C. Santiago C. D. Wadsworth M. E. (2009). Poverty and involuntary engagement stress responses: examining the link to anxiety and aggression within low-income families. Anxiety Stress Coping 22, 309–325. doi: 10.1080/10615800802430933, PMID: 19253173

[ref121] World Health Organization. (2020). WHO director-General’s opening remarks at the media briefing on COVID-19 March 11, 2020. Available at: https://www.who.int/dg/speeches/detail/who-director-general-s-opening-remarks-at-the-media-briefing-on-covid-19%2D%2D-11-march-2020 (accessed 13 October 2022).

[ref122] Worldometers (2022). Coronavirus death toll. Available at: https://www.worldometers.info/coronavirus/coronavirus-death-toll/ (accessed 15 November 2022).

[ref123] Xie X. Xue Q. Zhou Y. Zhu K. Liu Q. Zhang J.. (2020). Mental health status among children in home confinement during the coronavirus disease 2019 outbreak in Hubei Province China. JAMA Pediatrics 174, 898–900. doi: 10.1001/jamapediatrics.2020.1619, PMID: 32329784 PMC7182958

